# Effects of Pulsatile and Non-Pulsatile Cardiopulmonary Bypass on Early Inflammatory Response After Cardiac Surgery: A Secondary Biomarker Analysis of a Multicentric Randomized Controlled Trial

**DOI:** 10.3390/jcm15187228

**Published:** 2026-09-17

**Authors:** Aleksandra Ljubačev, Antonijo Grčić, Lara Batičić, Matej Jenko, Gordana Taleska Štupica, Božena Ćurko-Cofek, Mia Šestan, Gordana Laškarin, Danijel Knežević, Marino Damić, Vlatka Vujnović Đukić, Igor Medved, Maja Šoštarič, Miha Antonič, Marko Zdravković, Vlatka Sotošek

**Affiliations:** 1 Department of Surgery, Faculty of Medicine, University of Rijeka, Braće Branchetta 20, 51000 Rijeka, Croatia; aleksandra.ljubacev@uniri.hr (A.L.); igor.medved@uniri.hr (I.M.); 2 Department of Medical Chemistry, Biochemistry and Clinical Chemistry, Faculty of Medicine, University of Rijeka, Braće Branchetta 20, 51000 Rijeka, Croatia; antonijo.grcic@uniri.hr; 3 Clinical Department of Anesthesiology and Surgical Intensive Care, University Medical Centre Ljubljana, Zaloška Cesta 7, 1000 Ljubljana, Slovenia; matej.jenko@kclj.si (M.J.); taleskagordana@gmail.com (G.T.Š.); maja.sostaric@kclj.si (M.Š.); 4 Department of Anesthesiology and Reanimatology, Faculty of Medicine, University of Ljubljana, Vrazov Trg 2, 1000 Ljubljana, Slovenia; markozdravkovic@gmail.com; 5 Department of Physiology, Immunology and Pathophysiology, Faculty of Medicine, University of Rijeka, Braće Branchetta 20, 51000 Rijeka, Croatia; gordana.laskarin@uniri.hr; 6 Clinic of Anesthesiology, Intensive Medicine and Pain Management, Clinical Hospital Centre Rijeka, Krešimirova 42, 51000 Rijeka, Croatia; mia.sestan1@gmail.com (M.Š.); vlatkavd24@gmail.com (V.V.Đ.); vlatkast@uniri.hr (V.S.); 7 Hospital for Medical Rehabilitation of Hearth and Lung Diseases and Rheumatism “Thalassotherapia-Opatija”, M. Tita 188, 51410 Opatija, Croatia; 8 Department of Anesthesiology, Reanimatology, Emergency and Intensive Care Medicine, University of Rijeka, Braće Branchetta 20, 51000 Rijeka, Croatia; 9 Department for Cardiac Surgery, University Medical Centre Maribor, Ljubljanska Ulica 5, 2000 Maribor, Slovenia; miha.antonic@guest.arnes.si; 10 Faculty of Medicine, University of Maribor, Taborska 8, 2000 Maribor, Slovenia; 11 Department of Anesthesiology, Intensive Care and Pain Management, University Medical Centre Maribor, Ljubljanska Ulica 5, 2000 Maribor, Slovenia; 12 Department of Clinical Medical Sciences II, Faculty of Health Studies, University of Rijeka, Viktora Cara Emina 2, 51000 Rijeka, Croatia

**Keywords:** cardiac surgery, cardiopulmonary bypass, interleukin-1β, systemic inflammatory response, interleukin-18, perfusion, pulsatile, non-pulsatile

## Abstract

**Background/Objectives:** Cardiopulmonary bypass (CPB) induces a systemic inflammatory response that may contribute to postoperative organ dysfunction. However, the effects of pulsatile and non-pulsatile CPB on the inflammatory response remain incompletely understood. This study investigated the perioperative dynamics of interleukin (IL)-1β, IL-18, and IL-18 binding protein (IL-18BP), as pre-specified secondary inflammatory biomarker analysis of a prospective, multicentric, randomized trial (ISRCTN11243508), in patients undergoing cardiac surgery with pulsatile or non-pulsatile CPB and evaluated their relationships with routine inflammatory and myocardial injury biomarkers. **Methods:** In this study 142 patients undergoing elective cardiac surgery were randomized to pulsatile (Group P) or non-pulsatile (Group NP) CPB, and 129 were analyzed (pulsatile, *n* = 63; non-pulsatile, *n* = 66). Blood samples were collected before surgery and at five postoperative time points. Plasma concentrations of IL-1β, IL-18, and IL-18BP were measured by ELISA. Leukocyte count, C-reactive protein (CRP), procalcitonin (PCT), high-sensitivity cardiac troponin I (hsTnI), and N-terminal pro-B-type natriuretic peptide (NT-proBNP) were also analyzed. The trial was powered for its primary outcome; for these secondary outcomes the achieved sample size had 80% power to detect between-group ratios of geometric means of approximately 1.27 (IL-18), 1.41 (IL-18BP) and 1.38 (IL-1β). **Results:** Cardiac surgery with CPB induced a marked postoperative inflammatory response in both groups: leukocyte count, CRP, PCT, IL-18, IL-18BP, hsTnI and NT-proBNP increased significantly after surgery, whereas IL-1β remained essentially unchanged. In the pre-specified confirmatory comparison of postoperative peak concentrations, no biomarker differed significantly between groups. The 95% confidence intervals of this comparison exclude a pulsatile-flow effect on peak IL-18 larger than a 15% reduction or 19% increase, and on peak IL-18BP larger than a 26% reduction or 20% increase. A supporting mixed model for repeated measures showed no differential trajectory over time for any biomarker; its confidence intervals for the average postoperative effect were narrower (approximately ±10% for IL-18 and −9% to +15% for IL-18BP). The only exception was the leukocyte count, which was approximately 10% higher throughout the postoperative period in Group NP, without a differential trajectory shape. **Conclusions:** This pre-specified secondary analysis of a randomized trial found no evidence that pulsatile CPB reduces the early postoperative inflammatory or myocardial-injury response compared with non-pulsatile CPB for any of the eight biomarkers studied, apart from a modest difference in leukocyte count that did not survive correction for multiple comparisons. The confidence intervals obtained exclude only moderate-to-large between-group differences in IL-18 and IL-18BP; smaller true differences remain compatible with the data and cannot be ruled out at this sample size. These findings should not be interpreted as evidence of biochemical equivalence between pulsatile and non-pulsatile CPB, but they do not support a clinically important early anti-inflammatory advantage of pulsatile perfusion in this population.

## 1. Introduction

Cardiovascular diseases remain the leading global cause of mortality and morbidity, accounting for a substantial healthcare burden [1]. They are frequently treated surgically [2,3] using cardiopulmonary bypass (CPB), which provides a bloodless surgical field while ensuring adequate systemic perfusion and oxygenation. CPB can be conducted using either non-pulsatile or pulsatile blood flow.

Non-pulsatile CPB has continuous flow generated by roller or centrifugal pumps, which provide stable hemodynamics and simplified circuit management. During non-pulsatile CPB, a relatively constant mean arterial pressure (MAP) is maintained. However, the absence of physiological pulsatility reduces vascular wall shear stress and impairs mechanotransduction signaling pathways [4]. In contrast, pulsatile CPB aims to replicate the physiological hemodynamic characteristics. It generates dynamic biomechanical forces, including pulse pressure, cyclic shear stress, and cyclic vascular stretch, which are essential for maintaining endothelial homeostasis and microvascular function [5,6]. Pulsatility during CPB is achieved by modifying the arterial pump to create alternating phases of flow acceleration and deceleration, thereby simulating an arterial pulse with the goal of generating a minimum pulse pressure of 15 mmHg within the arterial line [7].

Regardless of the perfusion mode employed, cardiac surgery with CPB represents a highly invasive intervention associated with significant alterations in microcirculatory function [4]. Furthermore, CPB initiates a complex cascade of inflammatory and ischemia–reperfusion processes that contribute to acute tissue injury and may subsequently lead to postoperative organ dysfunction, thereby influencing both short- and long-term clinical outcomes [4,8,9].

Despite a growing interest in the biological effects of perfusion strategies, the impact of pulsatile and non-pulsatile CPB on the perioperative inflammatory response and cytokine release remains incompletely understood. Current clinical recommendations mention that pulsatile CPB may be used in adult cardiac surgical patients at increased risk of renal and pulmonary complications and those with longer CPB times—a class IIb recommendation [10,11,12]; however, robust evidence favoring one perfusion mode over the other is still lacking.

The inflammatory response associated with CPB is characterized by elevated circulating levels of leukocytes, C-reactive protein (CRP), procalcitonin (PCT) and early pro-inflammatory cytokines, including interleukin-1β (IL-1β) and interleukin-18 (IL-18), all of which have been implicated in the development of postoperative complications [8,13,14,15,16]. Following cardiac surgery, plasma IL-18 concentrations increase significantly and have been associated with both systemic inflammation and the development of acute kidney injury [17]. Additionally, experimental studies demonstrate that IL-18 induces cardiomyocyte hypertrophy [18].

The biological activity of IL-18 is tightly regulated by its antagonist IL-18 binding protein (IL-18BP). IL-18BP belongs to the immunoglobulin-like receptor family but is secreted rather than membrane-bound [19]. Experimental studies have demonstrated that IL-18BP attenuates myocardial injury following ischemia–reperfusion and reduces angiotensin II-induced cardiomyocyte hypertrophy [20,21]. Consequently, IL-18BP has emerged as a promising therapeutic target in autoimmune diseases and is increasingly being investigated for its cardioprotective potential in limiting myocardial injury and adverse remodeling [20].

Therefore, given the growing evidence supporting the important roles of IL-1β, IL-18, and IL-18BP in the inflammatory response, this study aimed to investigate the effects of pulsatile and non-pulsatile CPB on the perioperative dynamics of these biomarkers in patients undergoing open-heart surgery. A literature search of PubMed and Scopus identified no previous studies investigating their perioperative dynamics in this clinical setting. In addition, we assessed the correlations of IL-1β, IL-18, and IL-18BP with routinely used inflammatory markers, including CRP, PCT, and leukocyte count.

## 2. Materials and Methods

### 2.1. Patients

This prospective, multicentric, randomized study was approved by the Ethics Committee of the Clinical Hospital Centre Rijeka, Croatia (on 28 February 2023; Ref: 2170-29-02/1-23-2) and the Slovenian National Medical Ethics Committee (on 1 March 2024; Ref: 0120-33/2024-2711-3). The study was registered prior to patient enrollment at isrctn.com (ISRCTN11243508, Principal Investigator: Marko Zdravkovic, Date of registration: 21 May 2024). The study was conducted in accordance with the ethical principles of the Declaration of Helsinki and the guidelines of the World Medical Association. All participants received written study information and additional verbal explanations from the research team. Written informed consent was obtained prior to enrollment. The patients were recruited from three tertiary healthcare centers (Clinical Hospital Centre Rijeka, Croatia; University Medical Centre Ljubljana, Slovenia; and University Medical Centre Maribor, Slovenia) between 28 May 2024 and 8 June 2026. This manuscript adheres to the applicable CONSORT guidelines.

**Figure 1:**
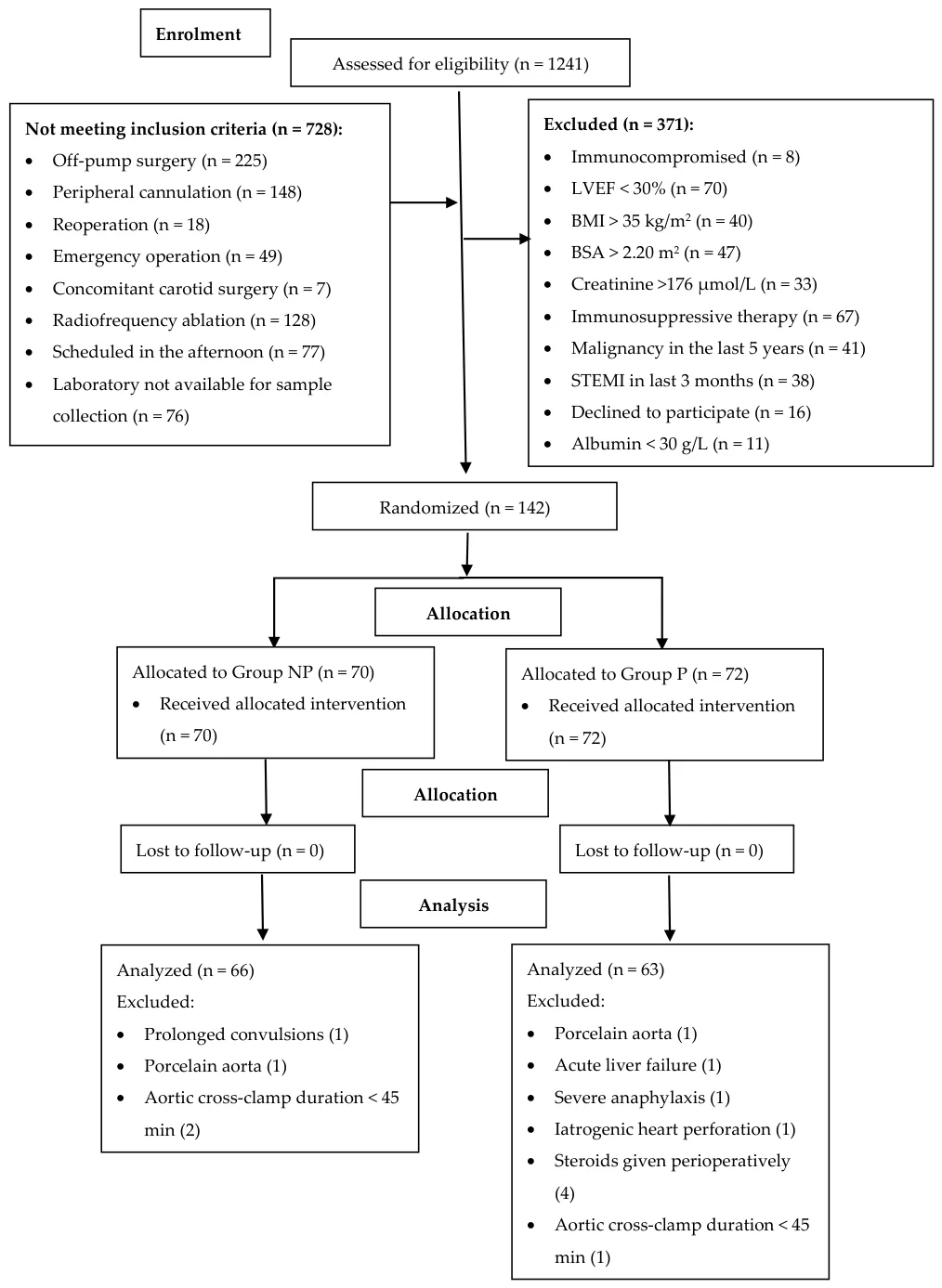
Flowchart of the study protocol. Group NP: patients who underwent non-pulsatile cardiopulmonary bypass; Group P: patients who underwent pulsatile cardiopulmonary bypass. LVEF—left ventricular ejection fraction; BMI—body mass index; BSA—body surface area; STEMI—ST elevation myocardial infarction.

Adult patients scheduled for elective cardiac surgery in the morning through median sternotomy with CPB and expected aortic cross-clamp duration of >45 min were assessed for eligibility [22]. Patients were allocated to either the non-pulsatile (Group NP) or pulsatile (Group P) CPB using institution-stratified permuted block randomization (Figure 1). Randomization was performed in a 1:1 ratio with a block size of six, using a computer-generated randomization sequence created with MedCalc Statistical Software version 20.014 (MedCalc Software Ltd., Ostend, Belgium). The allocated group was held in sequentially numbered opaque envelopes for each center until patients signed the informed consent. Patients were recruited by A.L. (Rijeka), M.J. and G.T.Š. (Ljubljana) and M.Z. (Maribor). Only patients and laboratory personnel were blinded to treatment allocation as the intervention was clear to surgeons and anesthesiologists. Each center was expected to recruit 48 patients, making a total of 144 patients with a 10% margin for subsequent exclusions due to major protocol violations (such as steroid administration, etc.).

Patients were excluded from the study if they refused to participate, had undergone repeat cardiac surgery, had a left ventricular ejection fraction (LVEF) < 30%, had a body mass index (BMI) > 35 kg/m^2^ or <18 kg/m^2^, had a body surface area > 2.20 m^2^, had advanced chronic lung disease (PaO_2_ on room air < 9.8 kPa), kidney disease (serum creatinine > 176 μmol/L), liver disease (albumin < 30 g/L and/or bilirubin > 34.2 μmol/L), an autoimmune disease, clinical and/or laboratory signs of infection, were receiving chemo/immunosuppressive therapy, active malignancy or a history of treatment for malignancy within the preceding 5 years, or had experienced an acute ischemic stroke or ST-elevation myocardial infarction within 3 months before surgery, known epilepsy or known allergy to any drugs used in the study protocol (Figure 1). After exclusions, 129 patients were included in this pre-specified secondary analysis of a randomized controlled trial (IL-1β, IL-18 and IL-18BP were registered secondary outcomes of the trial); *n* = 41 from Rijeka, 46 from Ljubljana and 42 from Maribor. An intention-to-treat analysis was not possible because sample collection and protocol treatment were discontinued in the excluded patients; the analysis population is therefore the pre-specified per-protocol population. For example, a porcelain aorta identified intraoperatively required a change in cannulation site and clamping strategy (e.g., axillary/femoral cannulation) that was incompatible with the standardized cannulation as defined for the trial, so the allocated CPB mode could not be delivered. In cases of aortic cross-clamp duration shorter than 45 min and severe anaphylaxis, there was likewise no clinical rationale to continue with the study protocol.

### 2.2. Anesthesia and Surgical Procedure

Common anesthesia, surgical and intensive care unit (ICU) management protocols were established for all three sites after a series of 12 multidisciplinary meetings, involving anesthesiologists, intensivists, cardiac surgeons and perfusionists from all three centers. Preoperative anxiolysis with oral midazolam/diazepam and other medications on the morning of surgery was administered as per each hospital protocol and the treating anesthesiologist’s decision. General balanced anesthesia during the procedure was achieved by intravenous administration of propofol, sufentanil and rocuronium bromide. All drug dosing was done as per the ideal body weight. No regional anesthesia techniques were allowed. Patients were endotracheally intubated and mechanically ventilated. Central venous catheters, urinary catheters and arterial lines for invasive blood pressure measurement and blood sampling were inserted. Fluid management before CPB was restrictive (i.e., <1000 mL of crystalloids). All patients received antibiotic prophylaxis and 20 mg/kg of tranexamic acid before surgical incision.

Patients were positioned supine and the surgical field was prepped. The surgical approach was done by full or partial median sternotomy and pericardiotomy. During the procedure, the CPB device was used (Stockert S5, Sorin Group Italia S.r.l., LivaNova PLC, Mirandola, Italy) with the hollow fiber membrane oxygenator (INSPIRE 8F PH.I.S.I.O., Sorin Group Italia S.r.l., LivaNova PLC, Mirandola, Italy) and a roller pump [23]. Priming solution volume was 1000–1250 mL and consisted of 250 mL 20% mannitol, 10.000 IU of heparin and Ringer solution (Ringer, B. Braun Melsungen AG, Melsungen, Germany). In all the patients, a straight 24F arterial cannula (EOPA™, Elongated One-Piece Arterial Cannula, Medtronic, Minneapolis, MN, USA) was used for standard cannulation of the ascending aorta [23]. Cardioplegic solutions were administered as per each institutional standard practice.

Systemic anticoagulation before commencement of the CPB was achieved by intravenous administration of 400 IU/kg of heparin. During CPB, the degree of systemic anticoagulation was monitored by sequential measurements of activated clotting time (ACT) with the maintenance of ACT values above 400 s. All the patients were operated in normothermia (35.0–37.0 °C of core body temperature).

Pulsatile CPB flow commenced after the placement of the aortic cross-clamp and ended after its release. The following parameters were used in the pulsatile CPB: frequency 75/min, flow width (percentage of pump time on maximum speed) 60%, base flow 30%. Minimum pulse pressure target was 15 mmHg [22,23]. Additional modifications were made considering the amplitude and slope of the pulse pressure curve. MAP between 55 and 80 mmHg and a cardiac index of 2.0–2.4 L/min/m^2^ were maintained. The duration of pulsatile CPB exposure corresponded to each patient’s individual aortic cross-clamp duration.

After successful CPB weaning, the effects of heparin were reversed in a 0.8–1.0 ratio by protamine sulphate infusion, and ACT values after reversion were checked. Following the completion of the surgical procedure, the patients were transferred to the cardiac ICU. When the conditions for emergence from anesthesia were achieved, extubation followed. Hemodynamically stable, spontaneously breathing patients were transferred to a step-down high-dependency unit at the department of cardiac surgery.

### 2.3. Blood Sample Collection

For each patient, arterial blood samples were collected at six time points: before induction of anesthesia (T1), 2 h (T2), 6 h (T3), 18 h (T4), 42 h (T5) and 66 h (T6) after CPB. Blood samples were sent for regular analyses (leucocytes, CRP, PCT and standard biochemical analysis) in the certified tertiary hospital laboratory at each site. Blood was also sampled in two 5 mL gel barrier tubes (VACUETTE^®^, REF 455083; Greiner Bio-One, Kremsmünster, Austria). Samples were delivered to the certified tertiary hospital laboratory within 30 min of collection and centrifuged immediately upon arrival at the laboratory. These samples were centrifuged at 3000 *g* for 10 min. Three plasma aliquots of more than 1 mL each were transferred into cryotubes and stored at −80 °C until further analysis of IL-1β, IL-18 and IL-18BP.

### 2.4. Measurement of Plasma Concentrations of IL-1β, IL-18 and IL-18BP

Plasma concentrations of IL-1β, IL-18 (Proteintech, Rosemont, IL, USA for both), and IL-18BP (Abcam, Cambridge, UK) were quantified in duplicates using commercially available high-sensitivity enzyme-linked immunosorbent assay (ELISA) kits according to the manufacturer’s instructions at a single central laboratory at the Faculty of Medicine, University of Rijeka, to minimize center-related pre-analytical variability.

Briefly, plasma samples and calibration standards were added to microplate wells pre-coated with analyte-specific antibodies and incubated under prescribed conditions. Following a series of washing steps to remove unbound material, a biotinylated detection antibody was applied, followed by incubation with streptavidin–horseradish peroxidase conjugate. Immunoreactivity was visualized by the addition of 3,3′,5,5′-tetramethylbenzidine substrate, and the enzymatic reaction was terminated using the supplied stop solution. Absorbance was measured using a microplate reader (EL808; BioTek Instruments, Winooski, VT, USA) at 450 nm with wavelength correction at 630 nm. Analyte concentrations were determined by interpolation from standard calibration curves generated for each assay and were expressed as pg/mL. Data processing and curve fitting were performed using dedicated ELISA analysis software (ElisaAnalysis; version 3.2; Elisakit Pty Ltd., Victoria, Australia).

To reduce inter-assay variability, all samples obtained from an individual participant were analyzed on the same ELISA plate. Laboratory personnel performing the analyses were blinded to treatment allocation throughout the study.

No ELISA measurement was below the assay detection or quantification limits; therefore, no data imputation, substitution, or censoring procedures were required for IL-1β, IL-18 or IL-18BP. Routine laboratory results reported at the lower reporting limit of the local laboratory (CRP 2 mg/L in 48%, 52%, and 9% of samples at T1, T2, and T3; PCT 0.02 µg/L in 39% and 6% of samples at T1 and T2; none thereafter) were analyzed at that limit. NT-proBNP was reported in pmol/L by the Maribor laboratory and was converted to ng/L (numerically equal to pg/mL) by multiplying by 8.457 (molar mass of NT-proBNP, 8457 g/mol) before analysis. Because hsTnI, PCT and NT-proBNP were measured with each site’s routine assay, recruiting center was included as a covariate in all longitudinal models (Section 2.5).

### 2.5. Statistical Analysis

Statistical analyses were performed with R version 4.5 (R Foundation for Statistical Computing, Vienna, Austria). The sample size of the parent trial was determined a priori for its primary outcome and is reported separately; the assumptions were derived from preliminary data of our group [24]. Because this is a pre-specified secondary analysis of a trial powered for a different outcome, no post hoc power calculation is reported (post hoc power is a function of the observed *p* value); instead, the smallest detectable difference at the achieved sample size is reported below.

The distributions of all eight biomarkers (IL-1β, IL-18, IL-18BP, leukocyte count, CRP, PCT, hsTnI and NT-proBNP) were right-skewed; all analyses were therefore performed on natural log-transformed concentrations. Every between-group effect is reported as the ratio of geometric means (Group P relative to Group NP), with its 95% confidence interval (CI) obtained by exponentiating the difference in means on the log scale. Results are interpreted primarily from the width and location of the CI rather than from the *p* value. Two complementary analyses were pre-specified: (i) a confirmatory comparison of the postoperative peak concentration of each biomarker between groups, and (ii) a supporting analysis of the full postoperative time-course.

For each biomarker, the peak postoperative concentration for each patient was defined as the maximum value observed at T2–T6. Biomarkers were grouped into two pre-specified families reflecting their role in this study. Family A (primary biomarkers of interest) comprised IL-1β, IL-18 and IL-18BP; Family B (secondary, routinely used biomarkers) comprised leukocyte count, CRP, PCT, hsTnI and NT-proBNP. Within each family, the log-transformed peak concentration was compared between Group P and Group NP using Welch’s *t*-test, which does not assume equal variances between groups; the Mann–Whitney U test on the untransformed peak values was performed as a sensitivity analysis. *p* values from Welch’s *t*-test were adjusted for multiple comparisons within each family using the Holm–Bonferroni procedure (over 3 comparisons for Family A and over 5 for Family B). This peak-value comparison is the pre-specified primary confirmatory test for a between-group difference in each biomarker.

For each biomarker, a single linear mixed-effects model was fitted to all post-CPB observations (2–66 h) with log-transformed concentration as the dependent variable, Group (NP vs. P), Time (T2–T6, categorical) and their interaction as fixed effects, log-transformed baseline (T1) concentration and recruiting center as covariates, and an unstructured covariance matrix for the five repeated measurements within a patient (mixed model for repeated measures), which makes no assumption about the correlation between time points. The within-group change over time is captured by the Time term of the model, and the between-group comparison by the Group and Group × Time terms, so that no separate non-parametric time-course test was additionally performed. The Group × Time interaction was tested with a Wald chi-square test on the four interaction terms. Time-point contrasts (Group P relative to Group NP at each postoperative time point) and the average between-group effect across the postoperative period (a single ratio of geometric means obtained from the corresponding model without the interaction term) are reported with t-based 95% CIs using 124 degrees of freedom. Models were fitted by restricted maximum likelihood using all available observations under a missing-at-random assumption; no imputation was performed. The number of missing observations for each biomarker, time point and group is reported in Supplementary Table S1.

Because the study was not independently powered for this secondary biomarker analysis, we report the smallest standardized between-group difference that the achieved sample size (66 patients in Group NP, 63 in Group P) could detect with 80% power at a two-sided α of 0.05. This corresponds to a Cohen’s d of 0.50 (0.58 at the Holm-adjusted α for a family of three comparisons). Applied to the observed variability of the log-transformed postoperative peak concentrations, the achieved sample size had 80% power to detect a ratio of geometric means of approximately 1.27 for IL-18, 1.41 for IL-18BP and 1.38 for IL-1β at the nominal α (1.32, 1.49 and 1.46, respectively, at the Holm-adjusted α); smaller true differences cannot be excluded by this study.

Baseline characteristics are presented descriptively, without hypothesis tests, as recommended by CONSORT. For the leukocyte count only, three post hoc, non-pre-specified patient-level summary measures (peak count, increment from baseline to peak, and time to peak) were compared between groups with the Mann–Whitney U test and, for time to peak, the chi-square test, as previously described; these are reported as exploratory and are not part of the confirmatory testing strategy above (Supplementary Table S2). Sensitivity analyses of the leukocyte model (an unadjusted mixed model and generalized estimating equations with robust standard errors) are given in Supplementary Table S3. All tests were two-sided. A Holm-adjusted *p* < 0.05 was considered statistically significant for the confirmatory Family A/B comparisons; for all other analyses unadjusted *p* values are reported and interpreted descriptively. Time-point contrasts between the groups from the mixed models for repeated measures are given in Supplementary Table S4.

Correlations between the three cytokines and the routine markers at each time point were assessed with Spearman’s rank correlation coefficient and are reported as exploratory (Supplementary Tables S5–S7).

Continuous variables are presented as median and interquartile range (IQR) unless otherwise stated.

## 3. Results

Demographic data, surgical data, and baseline biomarker concentrations are shown in Table 1. In Group P, a pulse pressure gradient of ≥15 mmHg was achieved and maintained throughout the entire duration of pulsatile CPB in all 63 patients.

### 3.1. Time Course of the Biomarkers

**Figure 2:**
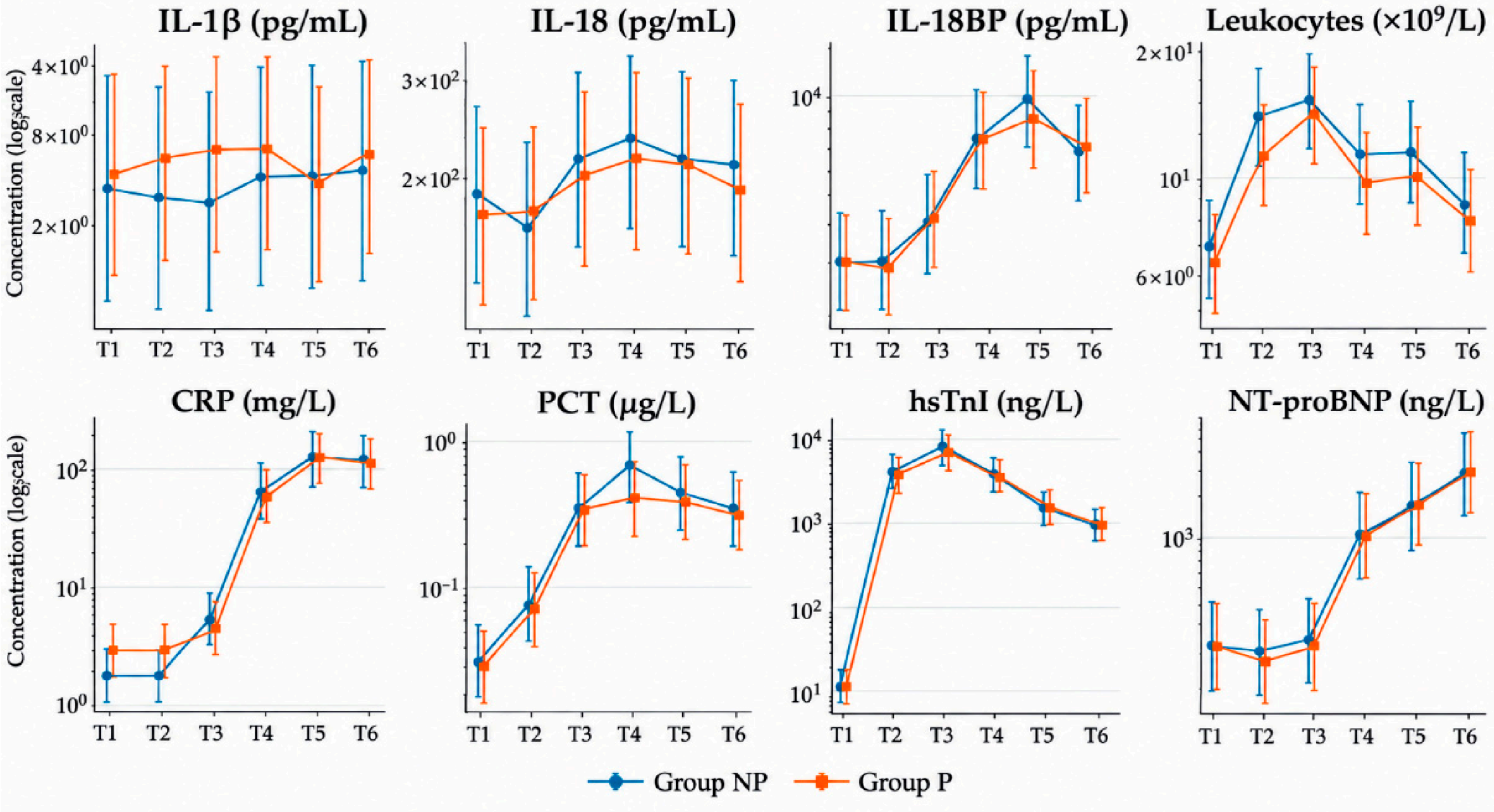
Time course of the eight biomarkers in the non-pulsatile (Group NP, *n* = 66) and pulsatile (Group P, *n* = 63) groups. Symbols show the median and the interquartile range at each time point before induction of anesthesia (T1) and 2 (T2), 6 (T3), 18 (T4), 42 (T5) and 66 (T6) hours after cardiopulmonary bypass on a logarithmic scale; symbols are offset horizontally for clarity. CRP—C-reactive protein; PCT—procalcitonin; hsTnI—high-sensitivity cardiac troponin I; NT-proBNP—N-terminal pro-B-type natriuretic peptide; IL—interleukin; BP—binding protein.

All biomarkers except IL-1β increased after surgery in both groups (Figure 2). Concentrations of IL-18 and IL-18BP increased from 6 h after CPB onward, whereas the concentration of IL-1β remained essentially unchanged (pooled medians 2.4–2.5 pg/mL), with only a small increase at time point T6 relative to baseline (ratio of geometric means 1.14, 95% CI 1.05–1.25). Among the routine markers, absolute number of leukocytes and hsTnI peaked at time points T2–T3, and concentrations of PCT, CRP and NT-proBNP reached their highest values at time points T4–T6.

### 3.2. Between-Group Comparison of Peak Concentrations

**Figure 3:**
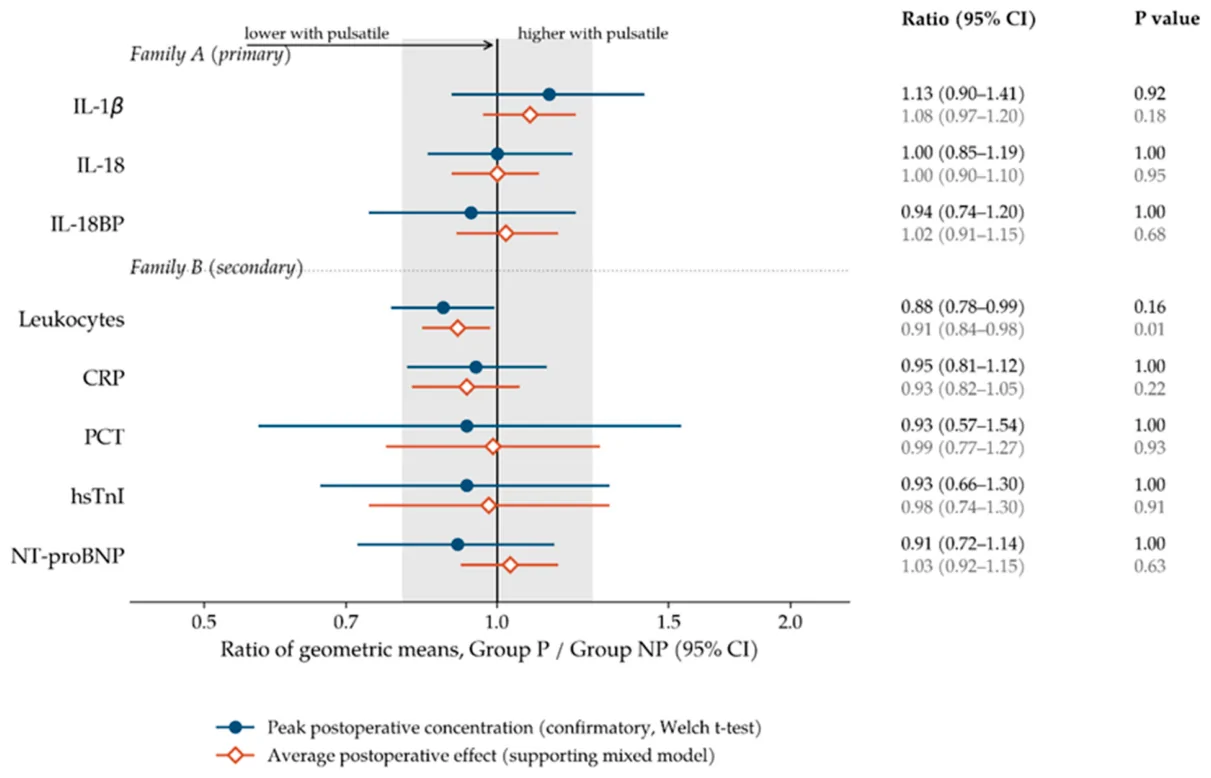
Between-group ratios of geometric means (Group P relative to Group NP) with 95% confidence intervals for the eight biomarkers. Filled circles: pre-specified confirmatory comparison of postoperative peak concentrations. Open diamonds: average between-group effect over the postoperative period from the mixed model for repeated measures. Values to the left of 1.0 indicate lower concentrations with pulsatile perfusion. The shaded band (0.80–1.25) is shown for orientation only; it was not pre-specified and does not represent a formal equivalence margin.

In the pre-specified confirmatory comparison, no biomarker differed between the groups after Holm adjustment (Table 2, Figure 3). For IL-18 and IL-18BP, the 95% CIs exclude an effect of pulsatile flow on the peak concentration larger than a 15% reduction or 19% increase, and larger than a 26% reduction or 20% increase, respectively. The peak leukocyte count was lower in Group P (ratio 0.88; Welch *p* = 0.032), but not after Holm adjustment (*p* = 0.16), and was followed by a gradual decline (Figure 2). Linear mixed-effects models for hsTnI and NT-proBNP did not provide evidence of a differential trajectory between groups (hsTnI: Wald χ^2^(4) = 5.99, *p* = 0.200; NT-proBNP: Wald χ^2^(4) = 3.06, *p* = 0.548), and the average adjusted effect over the postoperative period was likewise non-significant for hsTnI (1.016, 95% CI 0.767–1.345, *p* = 0.913) and NT-proBNP (0.973, 95% CI 0.872–1.086, *p* = 0.625) (Table 3, Supplementary Table S4).

### 3.3. Time-Course Analysis

The mixed model for repeated measures showed no group × time interaction for any biomarker (*p* ≥ 0.20; Table 3), and the shape of the postoperative trajectory did not differ between the groups. The average between-group effect over the postoperative period was close to 1 for all biomarkers except the absolute number of leukocytes, which was about 10% lower in Group P throughout the postoperative period (ratio 0.91, 95% CI 0.84–0.98; Table 3, Figure 3). This difference was largest at time point T2 (0.83, 95% CI 0.74–0.94). The absence of a group × time interaction was consistent across an unadjusted mixed model and generalized estimating equations (*p* = 0.28), whereas a joint test of any group difference over the postoperative period was borderline (*p* = 0.04–0.08 depending on covariate adjustment; Supplementary Table S3). Time-point contrasts for all biomarkers are given in Supplementary Table S4.

### 3.4. Exploratory Correlations

Spearman correlations between the concentrations of the cytokines, CRP, PCT, hsTnI, NT-proBNP and the absolute number of leukocytes at each time point were weak to moderate (|r| ≤ 0.54; 180 correlations, Supplementary Tables S5–S7). The most consistent were positive correlations between concentrations of IL-18BP and CRP or PCT in the later postoperative period in both groups. Concentrations of IL-1β correlated with concentrations of CRP and hsTnI at several time points in Group NP only. These associations are hypothesis-generating.

## 4. Discussion

This study provides a comprehensive investigation of the early inflammatory response [25,26] in patients following cardiac surgery with pulsatile or non-pulsatile CPB. We describe perioperative trends in plasma concentrations of currently used clinical markers of the inflammatory response (leukocytes, CRP, PCT), markers of myocardial injury (hsTnI, NT-proBNP) and three interleukins (IL-1β, IL-18 and IL-18BP) in both modes of CPB.

The dynamics of leukocyte activation observed in our study align with the well-established CPB-induced immune activation driven by blood–material contact, ischemia–reperfusion injury and surgical trauma [8,11]. Both groups showed a significant increase in the absolute number of leukocytes, peaking at 2–6 h after CPB followed by a gradual decline. The absolute number of leukocytes was approximately 10% higher in Group NP throughout the postoperative period, although this difference did not survive correction for multiple comparisons in the confirmatory peak-value comparison and should therefore be regarded as hypothesis-generating. Several methodological considerations may contribute to this observation. First, although total pump flow was maintained within an acceptable range of cardiac index of 2.0–2.4 L/min/m^2^, it was not standardized beyond routine perfusion practice and therefore variations in systemic blood flow could influence oxygen delivery, shear stress, and inflammatory signaling independently of flow pulsatility. Second, pulsatility generated by the extracorporeal pump may be substantially attenuated before reaching small vessel and capillary beds, the principal sites of leukocyte–endothelium interaction, which may have been exposed to a functionally non-pulsatile flow in both study groups. Third, the study population consisted predominantly of low-risk patients undergoing elective heart surgery, who generally exhibit less pronounced inflammatory response than higher-risk cardiac patients.

Previous studies evaluating inflammatory mediator release during cardiac surgery have reported inconsistent effects of pulsatile perfusion on the postoperative inflammatory response [27,28]. Difference in patient populations, perfusion systems, definitions of pulsatility, duration of pulsatile support, sampling schedules, and biomarker selection may contribute to the variability among studies. In addition, our recent review on cytokine response following open-heart surgery summarized IL-1β, IL-6, IL-8, tumor necrosis factor-alpha and IL-18 as key mediators involved in the postoperative inflammatory response with potential effects on leukocyte activation and leukocyte–endothelial interactions [29], lending further mechanistic plausibility to the interleukin kinetics observed in our cohort. The findings of the present study suggest that, in low-risk patients undergoing elective cardiac surgery, pulsatile CPB did not result in a consistent attenuation of circulating inflammatory cytokine concentrations compared with non-pulsatile CPB. Because this finding is isolated and CRP, PCT, and the cytokines did not differ between the groups, it requires further investigations; differences in hemodilution or fluid balance for example, which could also lower the leukocyte count, were not examined.

Plasma concentrations of CRP, PCT, hsTnI, and NT-proBNP exhibited temporal patterns consistent with their known kinetics following cardiac surgery with CPB [30]. CRP remained low during the early postoperative period and increased significantly postoperatively, peaking at 42–66 h post-CPB, reflecting its hepatic synthesis in response to IL-6 and other acute-phase stimuli. The hsTnI concentrations increased significantly at 2–6 h after CPB in both groups, consistent with perioperative myocardial injury, followed by a gradual decline. Cardioplegic arrest and ischemia–reperfusion are commonly related to an increase in hsTNI, but the surgical trauma of cardiac surgery itself, independent of ischemia–reperfusion, is also a major contributor to postoperative release of troponin [31]. NT-proBNP demonstrated a progressive postoperative increase reflecting ventricular wall stress and myocardial dysfunction during the early recovery period. None of these conventional biomarkers differed significantly between the groups, suggesting that the perfusion mode did not substantially alter the magnitude of myocardial injury or hemodynamic stress as detected by these markers. This observation aligns with findings from recent randomized trials showing no significant differences in troponin release or major clinical outcomes between pulsatile and non-pulsatile CPB in low-risk elective cardiac surgery populations [31].

Plasma concentration of IL-1β remained essentially unchanged over the perioperative period in both groups, despite marked leukocytosis and a rise in CRP. Since IL-1β acts predominantly in an autocrine and paracrine manner, has a short circulating half-life, and is rapidly neutralized by IL-1 receptor antagonist, its systemic concentrations may remain low even when local endothelial and myeloid activation is substantial [32,33].

Concentration of IL-18 and its endogenous antagonist IL-18BP both increased from 6 h after CPB onwards. The increase was parallel, and no statistically significant between-group difference was detected. IL-18BP binds mature IL-18 in a 1:1 complex with high affinity [19,34,35]. The concurrent increase in IL-18BP may represent an endogenous counter-regulatory response to increased IL-18 release. Because IL-18BP binds IL-18 with high affinity and prevents its interaction with the IL-18 receptor, measured total IL-18 does not necessarily reflect the amount of biologically active cytokine. Thus, simultaneous measurement of total IL-18 and IL-18BP could potentially be used to estimate the concentration of free, unbound IL-18 from [35]. Such an analysis could provide a more physiologically relevant measure of IL-18 activity than total IL-18 concentration alone. To our knowledge, this is one of the first randomized studies to examine perioperative changes in IL-18BP in patients undergoing CPB. The perioperative increase in IL-18BP suggests that regulatory mechanisms within the IL-18 pathway are activated after cardiac surgery and may help control the inflammatory response.

We analyzed correlations between all the markers to generate potential hypotheses for future studies. In the non-pulsatile group, IL-1β positively correlated with CRP and with hsTnI at several postoperative time points, whereas in the pulsatile group IL-1β showed almost no such coupling. Because IL-1β is generated largely at sites of endothelial and myeloid activation, this pattern is compatible with a closer link between early IL-1 activation and the systemic inflammatory and myocardial-injury response under non-pulsatile flow, and with a loosening of that link when pulsatility is preserved; however, these correlation coefficients were small (r ≤ 0.35), the between-group differences in correlation were not formally tested, and this pattern is hypothesis-generating. The absence of any between-group difference in IL-1β concentrations argues against a substantial effect of perfusion mode on IL-1β release itself [36,37,38]. The IL-18/IL-18BP axis, by contrast, behaved similarly under both perfusion modes. The association of IL-18 with CRP seen in the pulsatile group, and the isolated inverse associations of IL-18BP with the absolute number of leukocytes and of IL-18 with hsTnI were not consistent across time points or groups. The most consistent exploratory association was a positive correlation of IL-18BP with PCT and CRP in both groups, in line with the marked elevation of circulating IL-18BP reported in sepsis [35].

Previously studied biomarkers provide a useful comparison for the magnitude and timing of the response we observed [39,40,41]. It has been shown that implantation of a continuous-flow left ventricular assist device during CPB produces a marked but transient increase in IL-6, IL-8 and IL-10 concentrations that resolves within 24 h and is attributed mainly to the CPB rather than to the chronic non-pulsatile flow of the device itself [41].

Several limitations of this study should be acknowledged. Although the study population was carefully selected (i.e., for every 10 patients screened 1 was recruited), the clinical heterogeneity of patients undergoing cardiac surgery may have influenced the observed inflammatory responses. Furthermore, the relatively selected study population may limit the generalizability of the findings to higher-risk cardiac surgical patients. Differences in baseline characteristics, comorbid conditions, and types of surgical procedures could have contributed to variability in the results and represent potential confounding factors. Thirteen of the 142 randomized patients (9%) were excluded after randomization for pre-specified reasons, and an intention-to-treat analysis was not possible; the results therefore apply to the per-protocol population. Concentrations of hsTnI, PCT and NT-proBNP were measured with the routine assay of each center, which was included in all longitudinal models, but assay-related differences cannot be excluded. The study was powered for the primary outcome of the parent trial. For the biomarkers reported here, it could detect between-group differences of approximately 27–41%, so smaller differences cannot be excluded. Therefore, the absence of statistically significant differences should not be interpreted as evidence of biological equivalence. Future studies, including larger, even more homogeneous patient populations, are needed to better define the effects of non-pulsatile and pulsatile CPB on inflammatory responses. Another limitation is the relatively short follow-up period, which was focused on the early perioperative phase. Other mechanistic and clinical outcome analyses were beyond the scope of the present manuscript.

## 5. Conclusions

In this pre-specified secondary analysis of a randomized, multicentric trial, the mode of CPB did not produce a difference in any of the eight biomarkers, apart from a modest difference in the absolute number of leukocytes that did not withstand correction for multiple comparisons. Overall, this is a negative study. The confidence intervals obtained here exclude only a moderate-to-large effect of pulsatile flow on IL-18 and IL-18BP (larger than approximately a 15–26% reduction or 19–20% increase in the confirmatory peak comparison, and larger than approximately a 10% reduction or 10–15% increase for the average postoperative effect in the mixed model). Smaller true differences of a magnitude that could still be clinically relevant cannot be excluded.

This finding does not support a clinically important early anti-inflammatory advantage of pulsatile CPB over non-pulsatile CPB in this population, which is consistent with current guidelines that do not recommend routine use of pulsatile perfusion for this purpose. Likewise, this should not be read as proof of biochemical equivalence between the two modes.

The perioperative kinetics of IL-18 and IL-18BP described here, largely novel in this clinical setting, may still be useful for future studies. An adequately powered trial, designed specifically to test the biomarker hypotheses raised here, ideally in combination with markers of endothelial and complement activation, would be needed to resolve the residual uncertainty this study leaves open.

## Figures and Tables

**Table 1:** Baseline biomarker concentrations, demographic and surgical characteristics of the patients in non-pulsatile (Group NP) and pulsatile (Group P) groups of cardiopulmonary bypass (CPB).

	Group NP (*n* = 66)	Group P (*n* = 63)
Age (years)	64 (58–78)	64 (56–71)
Sex (male/female)	53/13	44/19
EuroSCORE II	1.21 (0.85–1.46)	1.15 (0.87–2.23)
Type of surgery		
CABG	22	22
Valve surgery	23	22
Combined procedures	21	19
Duration of CPB (min)	110 (91–139)	107 (85–128)
Aortic cross-clamp duration (min)	89.5 (68–108)	79 (66–102)
Duration of surgery (min)	229 (200–267)	225 (194–255)
Length of ICU stay (h)	24 (22–48)	24 (22–46)
Length of hospital stay (days)	8 (7–12)	8 (7–9.4)
In-hospital mortality (discharged/dead)	65/1	62/1
Baseline biomarker concentrations		
IL-1β (pg/mL)	2.28 (1.49–4.07)	2.42 (1.57–3.61)
IL-18 (pg/mL)	184 (140–236)	169 (139–236)
IL-18BP (pg/mL)	2585 (2038–3710)	2566 (1854–3562)
Leukocytes (×10^9^/L)	6.77 (5.43–7.93)	6.30 (5.58–7.60)
CRP (mg/L)	2 (2–5)	3 (2–5)
PCT (µg/L)	0.03 (0.02–0.05)	0.02 (0.02–0.05)
hsTnI (ng/L)	10.4 (7.0–21.0)	11.0 (6.1–19.0)
NT-proBNP (ng/L)	331 (147–675)	314 (120–819)

Continuous variables are presented as median and 25th–75th percentiles; categorical data are presented as the number of cases (*n*). CABG—coronary-artery bypass grafting; CRP—C-reactive protein; PCT—procalcitonin; EUROSCORE II—European System for Cardiac Operative Risk Evaluation II; ICU—intensive care unit; hsTnI—high-sensitivity cardiac troponin I; NT-proBNP—N-terminal pro-B-type natriuretic peptide; IL—interleukin; BP—binding protein.

**Table 2:** Pre-specified confirmatory comparison of postoperative peak concentrations between the pulsatile (Group P, *n* = 63) and non-pulsatile (Group NP, *n* = 66) groups.

Biomarker	Ratio of Geometric Means (P/NP)	95% CI	Welch *p*	Holm-Adjusted *p*	Mann–Whitney *p*
Family A (primary)					
Concentration of IL-1β (pg/mL)	1.13	0.90–1.41	0.31	0.92	0.42
Concentration of IL-18 (pg/mL)	1.00	0.85–1.19	0.96	1.00	0.84
Concentration of IL-18BP (pg/mL)	0.94	0.74–1.20	0.61	1.00	0.56
Family B (secondary)					
Absolute number of leukocytes (×10^9^/L)	0.88	0.78–0.99	0.032	0.16	0.007
Concentration of CRP (mg/L)	0.95	0.81–1.12	0.53	1.00	0.20
Concentration of PCT (µg/L)	0.93	0.57–1.54	0.79	1.00	0.85
Concentration of hsTnI (ng/L)	0.93	0.66–1.30	0.67	1.00	0.45
Concentration of NT-proBNP (ng/L)	0.91	0.72–1.14	0.40	1.00	0.56

Peak: highest value at T2–T6 for each patient. Ratios of geometric means > 1 indicate higher concentrations with pulsatile perfusion; Welch’s *t*-test on log-transformed values, *p* values Holm-adjusted within Family A (three comparisons) and Family B (five comparisons); the Mann–Whitney U test is a sensitivity analysis. CI—confidence interval.

**Table 3:** Mixed model for repeated measures: average between-group effect over the postoperative period (T2–T6) and test of the group × time interaction.

Biomarker	Average Ratio of Geometric Means (P/NP)	95% CI	*p*	Group × Time Interaction *p*
Concentration of IL-1β (pg/mL)	1.08	0.97–1.20	0.18	0.38
Concentration of IL-18 (pg/mL)	1.00	0.90–1.10	0.95	0.76
Concentration of IL-18BP (pg/mL)	1.02	0.91–1.15	0.68	0.87
Absolute number o leukocytes (×10^9^/L)	0.91	0.84–0.98	0.012	0.28
Concentration of CRP (mg/L)	0.93	0.82–1.05	0.22	0.45
Concentration of PCT (µg/L)	0.99	0.77–1.27	0.93	0.94
Concentration of hsTnI (ng/L)	0.98	0.74–1.30	0.91	0.20
Concentration of NT-proBNP (ng/L)	1.03	0.92–1.15	0.63	0.55

Ratios (Group P relative to Group NP) from the model without the interaction term, adjusted for the baseline concentration and recruiting center; values > 1 indicate higher concentrations with pulsatile perfusion. The interaction was tested with a Wald test on the four group × time terms.

## Data Availability

Data are available upon request.

## Supplementary Materials

The following supporting information can be downloaded at: https://www.mdpi.com/article/10.3390/jcm15187228/s1, Table S1. Number of missing observations per biomarker and time point, given as Group NP/Group P (*n* = 66 and *n* = 63). No imputation was performed; the mixed model for repeated measures uses all available observations and is valid under a missing-at-random assumption. Table S2. Post-hoc summary measures of the leukocyte response (exploratory). Patients contributed if a baseline value and at least four of the five postoperative values were available (Group NP, *n* = 66, Group P *n* = 63). Groups were compared with the Mann–Whitney U test, and the distribution of the time of peak with a chi-square test. Table S3. Sensitivity analyses of the mixed model for the absolute number of leukocytes. Two hypotheses are tested in each model: the group × time interaction (4 df; does the shape of the postoperative trajectory differ between the groups) and the joint hypothesis of no group difference at any postoperative time point (group and group × time terms together, 5 df). The pre-specified model (mixed model for repeated measures, unstructured covariance, adjusted for the baseline count and recruiting center) is compared with an unadjusted mixed model and with generalized estimating equations (GEE) with robust standard errors under two working correlation structures; the GEE models included center but not the baseline count. Table S4. Time-point contrasts between the groups from the mixed models for repeated measures (log-scale contrasts, back-transformed): ratio of geometric means (Group P relative to Group NP) at each postoperative time point with 95% confidence intervals and Wald test *p* values. Models are adjusted for the baseline (T1) concentration and recruiting center; these contrasts are supporting, hypothesis-generating detail and were not used to declare significance (see Table 3 of the main text for the average effect and the group × time interaction). Table S5. Correlations between IL-1β concentration and leukocyte count, CRP, PCT, hsTnI, and NT-proBNP concentrations in Groups NP and P at different time points: before induction of anesthesia (T1), 2 h (T2), 6 h (T3), 18 h (T4), 42 h (T5), and 66 h (T6) after cardiopulmonary bypass. Table S6. Correlations between IL-18 concentration and leukocyte count, CRP, PCT, hsTnI, and NT-proBNP concentrations in Groups NP and P at different time points: before induction of anesthesia (T1), 2 h (T2), 6 h (T3), 18 h (T4), 42 h (T5), and 66 h (T6) after cardiopulmonary bypass. Table S7. Correlations between IL-18BP concentration and leukocyte count, CRP, PCT, hsTnI, and NT-proBNP concentrations in Groups NP and P at different time points: before induction of anesthesia (T1), 2 h (T2), 6 h (T3), 18 h (T4), 42 h (T5), and 66 h (T6) after cardiopulmonary bypass.

## Abbreviations

The following abbreviations are used in this manuscript:

ACT	Activated clotting time
BMI	Body mass index
BSA	Body surface area
CABG	Coronary-artery bypass grafting
CPB	Cardiopulmonary bypass
CRP	C-reactive protein
ELISA	Enzyme-linked immunosorbent assay
EUROSCORE II	European System for Cardiac Operative Risk Evaluation II
Group P	patients receiving pulsatile cardiopulmonary bypass
Group NP	patients receiving non-pulsatile cardiopulmonary bypass
hsTnI	High-sensitivity cardiac troponin I
ICU	Intensive care unit
IQR	Interquartile range
IE	International units
IL-1β	Interleukin-1β
IL-18	Interleukin-18
IL-18BP	Interleukin-18 binding protein
LVEF	Left ventricular ejection fraction
MAP	Mean arterial pressure
*n*	number
NT-proBNP	N-terminal pro-B-type natriuretic peptide
*p*	level of statistical significance
PCT	Procalcitonin
r	correlation coefficient
