# Supplementary material for: Effects of Pulsatile and Non-Pulsatile Cardiopulmonary Bypass on Early Inflammatory Response After Cardiac Surgery: A Secondary Biomarker Analysis of a Multicentric Randomized Controlled Trial
Source: J Clin Med. 2026 Sep 17;15(18):7228. doi: 10.3390/jcm15187228 (PMC13607609; doi:10.3390/jcm15187228)
Supplement: Supplementary file 1 [file jcm-15-07228-s001.zip › jcm-4499344-supplementary.pdf]

Supplementary Materials

All between-group ratios in these tables are ratios of geometric means for the pulsatile group relative to the non-pulsatile group (Group P/Group NP); values above 1 indicate higher concentrations with pulsatile perfusion. T1 = before induction of anesthesia; T2–T6 = 2, 6, 18, 42 and 66 h after cardiopulmonary bypass.

Table S1. Number of missing observations per biomarker and time point, given as Group NP / Group P (n = 66 and n = 63). No imputation was performed; the mixed model for repeated measures uses all available observations and is valid under a missing-at-random assumption.

Biomarker	T1	T2	T3	T4	T5	T6
**Concentration of IL-1β (pg/mL)**	0 / 0	0 / 0	0 / 0	0 / 0	1 / 2	1 / 2
**Concentration of IL-18 (pg/mL)**	0 / 0	0 / 0	1 / 0	0 / 0	1 / 2	1 / 2
**Concentration of IL-18BP (pg/mL)**	0 / 0	0 / 0	0 / 0	1 / 0	1 / 2	1 / 2
**Absolute number of leukocytes**	0 / 0	0 / 2	0 / 0	0 / 0	0 / 0	1 / 0
**Concentration of CRP (mg/L)**	1 / 0	1 / 0	0 / 0	0 / 0	2 / 0	1 / 0
**Concentration of PCT (μg/L)**	0 / 0	0 / 2	1 / 0	0 / 1	3 / 0	2 / 0
**Concentration of hsTnI (ng/L)**	0 / 0	0 / 1	0 / 1	0 / 0	1 / 0	1 / 0
**Concentration of NT-proBNP (ng/L)**	0 / 0	1 / 1	0 / 0	0 / 0	2 / 0	1 / 0

Table S2. Post-hoc summary measures of the leukocyte response (exploratory). Patients contributed if a baseline value and at least four of the five postoperative values were available (Group NP n = 66, Group P n = 63). Groups were compared with the Mann–Whitney U test, and the distribution of the time of peak with a chi-square test.

Measure	Group NP	Group P	P
**Peak postoperative leukocyte count (×10⁹/L), median (IQR)**	16.32 (13.60–19.08)	13.98 (11.75–16.78)	0.007
**Increment from baseline to peak (×10⁹/L), median (IQR)**	9.22 (7.45–12.03)	7.90 (5.25–9.53)	0.007
**Peak-to-baseline ratio, median**	2.47	2.31	0.064
**Time of peak: T2 / T3 / T4 / T5 / T6, n**	14 / 40 / 6 / 5 / 1	10 / 37 / 6 / 9 / 1	0.762

Table S3. Sensitivity analyses of the mixed model for the absolute number of leukocytes. Two hypotheses are tested in each model: the group × time interaction (4 df; does the shape of the postoperative trajectory differ between the groups) and the joint hypothesis of no group difference at any postoperative time point (group and group × time terms together, 5 df). The pre-specified model (mixed model for repeated measures, unstructured covariance, adjusted for the baseline count and recruiting center) is compared with an unadjusted mixed model and with generalized estimating equations (GEE) with robust standard errors under two working correlation structures; the GEE models included center but not the baseline count.

Model	Group × time interaction (4 df)	Any group difference: group + group × time (5 df)
**Mixed model for repeated measures, adjusted (pre-specified)**	$\chi^2(4) = 5.09$, P = 0.279	$\chi^2(5) = 11.64$, P = 0.040
**Mixed model for repeated measures, unadjusted**	$\chi^2(4) = 5.04$, P = 0.283	$\chi^2(5) = 9.92$, P = 0.078
**GEE, exchangeable working correlation**	$\chi^2(4) = 5.15$, P = 0.272	$\chi^2(5) = 9.89$, P = 0.079
**GEE, independence working correlation**	$\chi^2(4) = 5.31$, P = 0.257	$\chi^2(5) = 10.59$, P = 0.060

Wald tests. GEE – generalized estimating equations.

Table S4. Time-point contrasts between the groups from the mixed models for repeated measures (log-scale contrasts, back-transformed): ratio of geometric means (Group P relative to Group NP) at each postoperative time point with 95% confidence intervals and Wald test P values. Models are adjusted for the baseline (T1) concentration and recruiting center; these contrasts are supporting, hypothesis-generating detail and were not used to declare significance (see Table 3 of the main text for the average effect and the group × time interaction).

Biomarker	Time	Ratio (P/NP)	95% CI	P
**Concentration of IL-1β (pg/mL)**	T2	1.18	1.00–1.40	0.057
	T3	1.04	0.89–1.23	0.607
	T4	1.07	0.92–1.24	0.374
	T5	1.04	0.92–1.17	0.564
	T6	1.11	0.94–1.31	0.222
**Concentration of IL-18 (pg/mL)**	T2	1.01	0.90–1.15	0.822
	T3	0.98	0.87–1.10	0.687
	T4	1.00	0.89–1.13	0.990
	T5	1.04	0.90–1.21	0.567
	T6	1.02	0.89–1.18	0.728
**Concentration ofIL-18BP (pg/mL)**	T2	1.01	0.87–1.18	0.858
	T3	0.98	0.85–1.13	0.793
	T4	1.01	0.83–1.23	0.919
	T5	1.03	0.84–1.27	0.770
	T6	1.08	0.92–1.28	0.337
**Absolute number of leukocytes (×10^9/L)**	T2	0.83	0.74–0.94	0.003
	T3	0.92	0.81–1.04	0.179
	T4	0.94	0.85–1.04	0.208
	T5	0.91	0.82–1.02	0.112
	T6	0.93	0.84–1.03	0.175
**Concentration ofCRP (mg/L)**	T2	1.12	0.86–1.45	0.405
	T3	0.87	0.68–1.11	0.269
	T4	0.87	0.74–1.01	0.066
	T5	0.88	0.74–1.05	0.169
	T6	0.93	0.77–1.12	0.422
**Concentration of PCT (μg/L)**	T2	0.97	0.74–1.28	0.844
	T3	0.97	0.66–1.44	0.882
	T4	0.93	0.60–1.43	0.735
	T5	1.01	0.66–1.53	0.980
	T6	0.96	0.64–1.46	0.864
**Concentration of hsTnI (ng/L)**	T2	1.01	0.74–1.39	0.935
	T3	1.04	0.76–1.41	0.808
	T4	1.03	0.73–1.45	0.856
	T5	1.09	0.74–1.61	0.654
	T6	0.93	0.62–1.38	0.701
**Concentration of NT-proBNP (ng/L)**	T2	1.08	0.94–1.25	0.269
	T3	1.08	0.90–1.28	0.410
	T4	0.93	0.74–1.16	0.516
	T5	0.95	0.73–1.25	0.721
	T6	0.87	0.64–1.19	0.386

CI – confidence interval; CRP – C-reactive protein; PCT – procalcitonin; hsTnI – high-sensitivity cardiac troponin I; NT-proBNP – N-terminal pro-B-type natriuretic peptide; IL – interleukin; BP – binding protein.

Table S5. Correlations between IL-1β concentration and leukocyte count, CRP, PCT, hsTnI, and NT-proBNP concentrations in Groups NP and P at different time points: before induction of anesthesia (T1), 2 hours (T2), 6 hours (T3), 18 hours (T4), 42 hours (T5), and 66 hours (T6) after cardiopulmonary bypass

Correlations		Absolute number of leukocytes (×10⁹/L)	Concentration of CRP (mg/L)	Concentration of PCT (μg/L)	Concentration of hsTnI (ng/L)	Concentration of NT-proBNP (ng/L)
**Concentration of IL-1β (pg/mL)**						
**Group NP (n = 66)**						
T1	*r*	0.094	**0.296**	0.171	-0.023	-0.096
	P	0.452	**0.017**	0.169	0.855	0.442
T2	*r*	-0.010	**0.276**	**0.257**	**0.277**	-0.096
	P	0.937	**0.026**	**0.038**	**0.024**	0.448
T3	*r*	-0.084	0.144	0.159	**0.262**	-0.100
	P	0.501	0.249	0.207	**0.033**	0.423
T4	*r*	-0.076	**0.283**	0.077	0.232	-0.012
	P	0.543	**0.021**	0.537	0.060	0.923
T5	*r*	0.036	**0.353**	0.061	0.244	-0.029
	P	0.775	**0.004**	0.635	0.051	0.820
T6	*r*	0.026	**0.291**	0.084	0.074	-0.099
	P	0.840	**0.020**	0.513	0.560	0.434
**Group P (n = 63)**						
T1	*r*	0.120	0.058	-0.002	-0.036	-0.086
	P	0.351	0.652	0.987	0.777	0.505
T2	*r*	0.068	0.198	**0.266**	0.152	0.006
	P	0.604	0.120	**0.039**	0.237	0.962
T3	*r*	0.065	0.198	0.052	0.181	0.036
	P	0.610	0.120	0.685	0.160	0.781
T4	*r*	0.017	0.150	0.020	0.164	0.089
	P	0.895	0.240	0.876	0.200	0.489
T5	*r*	0.000	0.078	0.175	0.050	0.072
	P	0.999	0.549	0.178	0.704	0.584
T6	*r*	0.056	0.072	0.100	0.174	-0.127
	P	0.667	0.583	0.443	0.181	0.328

P -level of statistical significance, *r* – correlation coefficient

Table S6. Correlations between IL-18 concentration and leukocyte count, CRP, PCT, hsTnI, and NT-proBNP concentrations in Groups NP and P at different time points: before induction of anesthesia (T1), 2 hours (T2), 6 hours (T3), 18 hours (T4), 42 hours (T5), and 66 hours (T6) after cardiopulmonary bypass .

Correlations		Absolute number of leukocytes (×10⁹/L)	Concentration of CRP (mg/L)	Concentration of PCT (μg/L)	Concentration of hsTnI (ng/L)	Concentration of NT-proBNP (ng/L)
**Concentration of IL-18 (pg/mL)**						
**Group NP (n = 66)**						
T1	*r*	0.094	-0.165	0.095	0.051	-0.025

	P	0.454	0.190	0.446	0.683	0.844
T2	*r*	0.017	-0.007	-0.027	-0.170	0.136
	P	0.891	0.955	0.831	0.171	0.279
T3	*r*	0.036	-0.046	-0.168	**-0.285**	0.186
	P	0.777	0.718	0.186	**0.021**	0.137
T4	*r*	0.114	-0.011	0.028	0.088	0.269
	P	0.362	0.931	0.823	0.481	**0.029**
T5	*r*	**0.271**	0.243	0.017	0.037	0.153
	P	**0.029**	0.053	0.894	0.767	0.226
T6	*r*	**0.339**	0.169	0.059	0.152	0.099
	P	**0.006**	0.183	0.649	0.229	0.436
**Group P (n = 63)**						
T1	*r*	-0.116	0.064	-0.099	0.246	0.116
	P	0.364	0.620	0.439	0.052	0.367
T2	*r*	0.088	0.146	0.007	**-0.281**	0.090
	P	0.501	0.253	0.957	**0.027**	0.485
T3	*r*	-0.136	0.199	-0.026	-0.107	0.135
	P	0.287	0.118	0.842	0.408	0.293
T4	*r*	0.018	0.164	-0.061	-0.089	-0.066
	P	0.888	0.199	0.640	0.489	0.607
T5	*r*	0.035	**0.439**	0.114	0.108	0.168
	P	0.791	**<0.001**	0.380	0.407	0.197
T6	*r*	-0.017	**0.432**	0.074	0.184	0.101
	P	0.894	**0.001**	0.570	0.156	0.438

P -level of statistical significance, *r* – correlation coefficient

Table S7. Correlations between IL-18BP concentration and leukocyte count, CRP, PCT, hsTnI, and NT-proBNP concentrations in Groups NP and P at different time points: before induction of anesthesia (T1), 2 hours (T2), 6 hours (T3), 18 hours (T4), 42 hours (T5), and 66 hours (T6) after cardiopulmonary bypass .

**Correlations**		**Absolute number of leukocytes ($\times 10^9$/L)**	**Concentration of CRP (mg/L)**	**Concentration of PCT (μg/L)**	**Concentration of hsTnI (ng/L)**	**Concentration of NT-proBNP (ng/L)**
**Concentration of IL-18BP (pg/mL)**						
**Group NP (n = 66)**						
T1	*r*	-0.065	0.094	0.146	0.158	0.056
	P	0.603	0.457	0.241	0.205	0.655
T2	*r*	0.024	0.051	**0.280**	0.127	0.006
	P	0.851	0.687	**0.023**	0.309	0.962
T3	*r*	**-0.318**	0.112	**0.303**	0.139	0.032
	P	**0.009**	0.369	**0.014**	0.266	0.797
T4	*r*	**-0.324**	**0.245**	**0.507**	0.138	0.098
	P	**0.009**	**0.049**	**<0.001**	0.271	0.439
T5	*r*	-0.140	**0.542**	**0.272**	0.044	0.164
	P	0.268	**<0.001**	**0.032**	0.730	0.194
T6	*r*	0.020	**0.301**	0.023	-0.012	0.135
	P	0.878	**0.016**	0.856	0.926	0.287

**Group P (n = 63)**						
T1	*r*	-0.008	-0.009	0.214	0.069	0.085
	P	0.948	0.944	0.092	0.593	0.506
T2	*r*	0.226	0.170	**0.366**	0.170	0.214
	P	0.079	0.182	**0.004**	0.186	0.095
T3	*r*	0.186	0.237	**0.460**	-0.016	0.062
	P	0.143	0.062	**<0.001**	0.903	0.629
T4	*r*	0.050	0.193	**0.277**	0.234	0.206
	P	0.697	0.129	**0.030**	0.065	0.105
T5	*r*	-0.023	**0.278**	0.146	0.252	0.125
	P	0.862	**0.030**	0.261	0.050	0.337
T6	*r*	-0.113	0.168	0.078	0.147	0.152
	P	0.386	0.195	0.551	0.257	0.241

P -level of statistical significance, *r* – correlation coefficient
